# *Chlamydia trachomatis* Requires Functional Host-Cell Mitochondria and NADPH Oxidase 4/p38MAPK Signaling for Growth in Normoxia

**DOI:** 10.3389/fcimb.2022.902492

**Published:** 2022-05-26

**Authors:** Jeewan Thapa, Gen Yoshiiri, Koki Ito, Torahiko Okubo, Shinji Nakamura, Yoshikazu Furuta, Hideaki Higashi, Hiroyuki Yamaguchi

**Affiliations:** ^1^ Division of Bioresources, International Institute for Zoonosis Control, Hokkaido University, Sapporo, Japan; ^2^ Department of Medical Laboratory Science, Faculty of Health Sciences, Hokkaido University, Sapporo, Japan; ^3^ Laboratory of Morphology and Image Analysis, Research Support Center, Juntendo University Graduate School of Medicine, Tokyo, Japan; ^4^ Division of Infection and Immunity, International Institute for Zoonosis Control, Hokkaido University, Sapporo, Japan

**Keywords:** *Chlamydia trachomatis*, mitochondria, normoxia, hypoxia, NADPH oxidase, NOX4, p38MAPK

## Abstract

*Chlamydia trachomatis* (Ct) is an intracellular energy-parasitic bacterium that requires ATP derived from infected cells for its growth. Meanwhile, depending on the O_2_ concentration, the host cells change their mode of ATP production between oxidative phosphorylation in mitochondria (Mt) and glycolysis; this change depends on signaling *via* reactive oxygen species (ROS) produced by NADPH oxidases (NOXs) as well as Mt. It has been proposed that Ct correspondingly switches its source of acquisition of ATP between host-cell Mt and glycolysis, but this has not been verified experimentally. In the present study, we assessed the roles of host-cell NOXs and Mt in the intracellular growth of CtL2 (L2 434/Bu) under normoxia (21% O_2_) and hypoxia (2% O_2_) by using several inhibitors of NOXs (or the downstream molecule) and Mt-dysfunctional (Mt^d^) HEp-2 cells. Under normoxia, diphenyleneiodonium, an inhibitor of ROS diffusion, abolished the growth of CtL2 and other Chlamydiae (CtD and *C. pneumoniae*). Both ML171 (a pan-NOX inhibitor) and GLX351322 (a NOX4-specific inhibitor) impaired the growth of CtL2 under normoxia, but not hypoxia. NOX4-knockdown cells diminished the bacterial growth. SB203580, an inhibitor of the NOX4-downstream molecule p38MAPK, also inhibited the growth of CtL2 under normoxia but not hypoxia. Furthermore, CtL2 failed to grow in Mt^d^ cells under normoxia, but no effect was observed under hypoxia. We conclude that under normoxia, Ct requires functional Mt in its host cells as an ATP source, and that this process requires NOX4/p38MAPK signaling in the host cells. In contrast to hypoxia, crosstalk between NOX4 and Mt *via* p38MAPK may be crucial for the growth of Ct under normoxia.

## Introduction

The obligate intracellular bacterium *Chlamydia trachomatis* (Ct), which is an energy parasite, is the leading cause of bacterial sexually transmitted infections, with an estimated 131 million new cases of infection annually worldwide (O’Connell and Ferone, 2016). The normal O_2_ concentration at the infection site is around 5% (Juul et al., 2007), and Ct also provokes an inflammatory response that consumes O_2_, resulting in hypoxia (Eltzschig and Carmeliet, 2011). Ct clearly favors hypoxia, and activates phosphatidylinositol-3 kinase (PI3K)/protein kinase B (AKT) in its host, which prompts glycolysis (Rupp et al., 2007; Thapa et al., 2020). However, Ct can grow well in host cells regardless of O_2_ conditions (Shima et al., 2011; Jerchel et al., 2014; Thapa et al., 2020).

Ct matures in infected host cells *via* a unique developmental cycle consisting of an infectious form (the elementary body, EB) and a replicating form (the reticular body) (Cossé et al., 2018). The successful maturation of Ct occurs in a customized plasma membrane, referred to as an inclusion body (Elwell et al., 2016); sufficient ATP is critical for the maturation. Because Ct possesses an incomplete tricarboxylic acid cycle (Tipples and McClarty, 1993; Harris et al., 2012), the maturation of Ct in the host cells absolutely relies on a stable supply of ATP derived from the host regardless of O_2_ conditions. However, the mechanism by which Ct acquires ATP from infected cells regardless of the O_2_ concentration is not well understood.

Mitochondria (Mt) are the main power plant in eukaryotic cells, responsible for generating ATP in an O_2_-dependent manner (Sousa et al., 2018). However, when O_2_ levels become low, the cells shift ATP production from Mt to glycolysis (Lunt and Vander Heiden, 2011). The molecular mechanism responsible for the switch is gradually becoming clear from research into cancer cells, and reactive oxygen species (ROS) generated by NADPH oxidases (NOXs) as well as Mt are a key factor (Kang et al., 2015; Phadwal et al., 2021). Specifically, studies have demonstrated that changes in the amount of ROS in the cells play a crucial role in switching cellular signals between p38MAPK signaling, which is responsible for the stabilization of Mt (Corbi et al., 2013), and AKT signaling, which is responsible for the activation of glycolysis (Xie et al., 2019; Kim et al., 2020; Vaupel and Multhoff, 2021). Furthermore, crosstalk between NOXs and Mt *via* p38MAPK has been proposed to be a crucial mechanism for prompting survival processes such as angiogenesis and tissue repair demanded more energy ATP (Fukai and Ushio-Fukai, 2020), which presumably also supports the intracellular growth of Ct under normoxia. However, the roles of Mt and NOXs in the intracellular growth of Ct and its response to O_2_ concentration and the associated signals have not yet been verified. Moreover, it has not yet been determined whether Ct in infected cells requires Mt as the site of ATP acquisition depending on O_2_ condition.

In the present study, we thus compared the roles of NOXs and Mt in the intracellular growth of Ct under normoxia (21% O_2_) and hypoxia (2% O_2_) by using Mt-dysfunctional (Mt^d^) human epithelial (HEp-2) cells and several inhibitors of NOXs and p38MAPK, which is a NOX4-related molecule.

## Results And Discussion

### Cytotoxicity of the Inhibitors Used, Expression Levels of NOXs, and the Effect of Diphenyleneiodonium on the Growth of Chlamydiae

**Figure 1:**
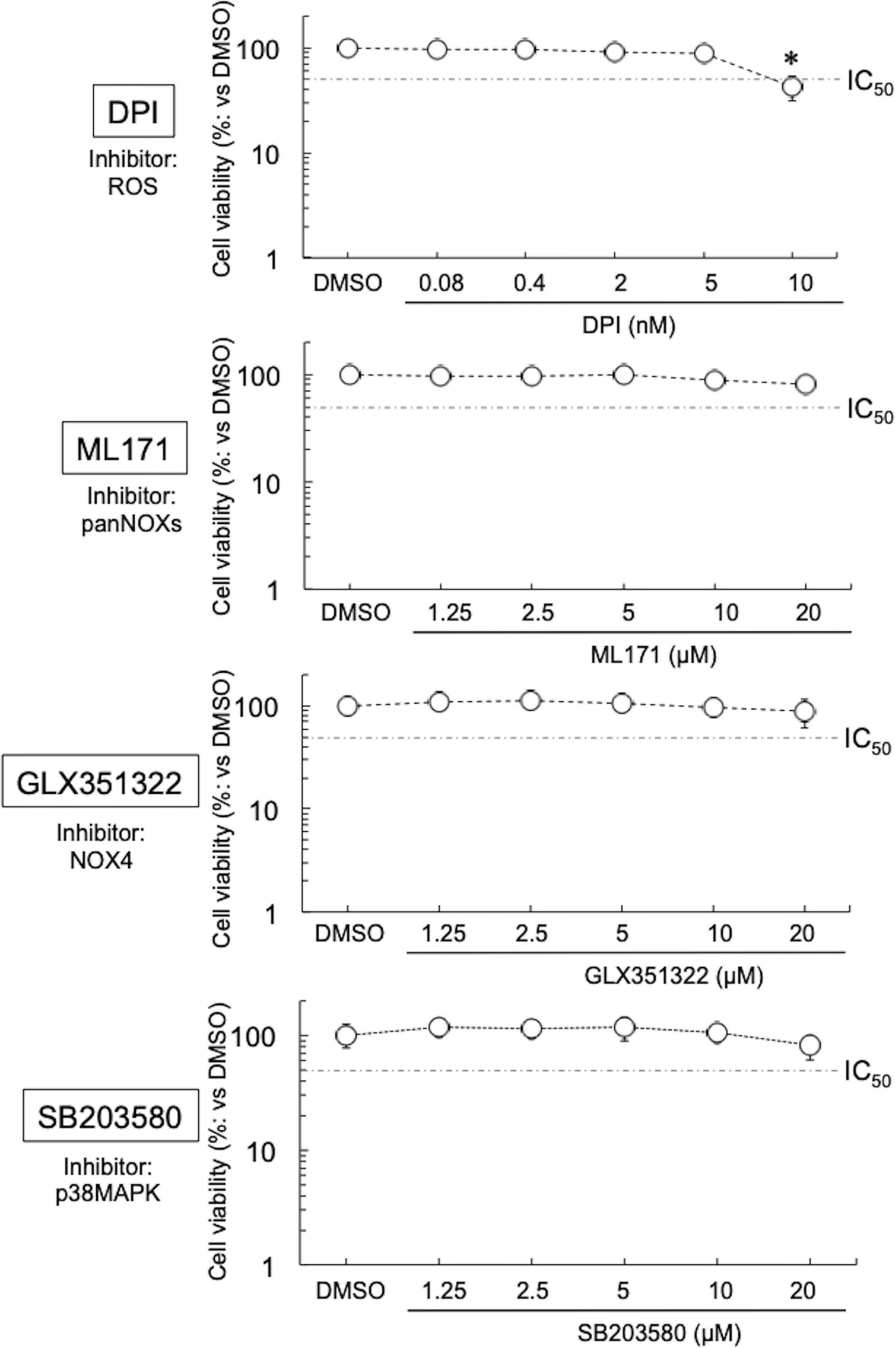
Viability of HEp-2 cells in the presence of inhibitors (DPI, ML171, GLX351322, and SB203580). The cells were cultured with or without inhibitor for 24 h, and then the cell viabilities were assessed using Cell Counting Kit-8 (see *Methods*). Data show means ± SD (*n* = 6) as a relative value of the cell-only (dimethylsulfoxide-treated) viability (which is defined as 100%). *p < 0.05 *vs*. dimethylsulfoxide-treated. Dotted lines show IC_50_ values.

Four inhibitors were used in this study—they block the diffusion of ROS (DPI) (Iacovino et al., 2020), or the activation of NOXs or related molecules (ML171, pan-NOXs; GLX351322, a NOX4 specific inhibitor; and SB203580, a p38MAPK-specific inhibitor) (Zhou et al., 2010; Cifuentes-Pagano et al., 2012; Anvari et al., 2015). No cytotoxicity of DPI, ML171, GLX351322, and SB203580 on HEp-2 cells was seen at ≤5 nM, ≤20 μM, ≤20 μM, and ≤20 μM, respectively (**Figure 1**). On the basis of these results, the inhibitors were used in our experiments at a concentration where no cytotoxicity was observed. Next, the expression levels of NOXs in the immortal human epithelial (HEp-2) cells used were examined by quantitative (q) reverse transcription (RT)-polymerase chain reaction (PCR). The expression level of NOX4 was the highest among the NOXs, at least 10 times that of the other NOXs (**Figure S1A**), indicating that, among NOXs, NOX4 could play the most important role in the growth of Ct. Furthermore, the effect of DPI on the growth of other chlamydiae [CtL2 (267, see *Methods*) and CtD (D/UW3/CX)] was assessed by using qPCR targeting the Ct *16S rDNA* gene. DPI significantly suppressed the growth of the Ct in a similar way (**Figure S1B**), indicating that Ct relies on ROS derived form NOXs for growth *via* a mechanism that might be conserved.

Although DPI at low concentrations suppressed the growth of various Chlamydiae, at ≥10 nM it showed strong cytotoxicity toward HEp-2 cells. DPI not only suppresses the activity of various NOXs (Iacovino et al., 2020) but also strongly inhibits the activities of cytochrome P-450 reductase (Chakraborty and Massey, 2002), xanthine oxidase (Sanders et al., 1997), nitric oxide synthase (Stuehr et al., 1991), and NADH-ubiquinone oxidoreductase (Majander et al., 1994). Overall, such pleiotropic effects are considered to be the cause of the strong cytotoxicity. Therefore, experiments with more selective inhibitors are needed to validate the role of NOXs in chlamydial intracellular growth.

As noted above, we found high expression of NOX4 in HEp-2 cells compared with that of other NOXs (**Figure S1B**). This result was consistent with the previous finding that, in contrast to other NOXs, NOX4 is highly expressed in cellular membrane in vascular cells or endothelial cells (Ago et al., 2004; Griendling, 2004). NOX4 is constitutively active, involving the control of cytoskeletal integrity (Lyle et al., 2009; Nisimoto, 2010), required for the growth of Ct. Furthermore, NOX4-dependent ROS is involved in many physiological functions, including immune host defense and the activation of multiple cellular signaling pathways such as SAPK/JNK, ERK1/2, and p38MAPK, which are responsible for ATP energy production *via* Mt) (Irani, 2000). Some of these pathways have already been reported to be targets of Ct (Shima et al, 2011; Jerchel et al., 2014; Thapa et al., 2020). Thus, we hypothesized that NOX4 is one of the targets of Ct for its successful intracellular growth under normoxia; if so, that would impact the production of ROS from NOXs as well as Mt.

### CtL2 Uses NOX4/p38MAPK Signaling for Its Growth Under Normoxia

**Figure 2:**
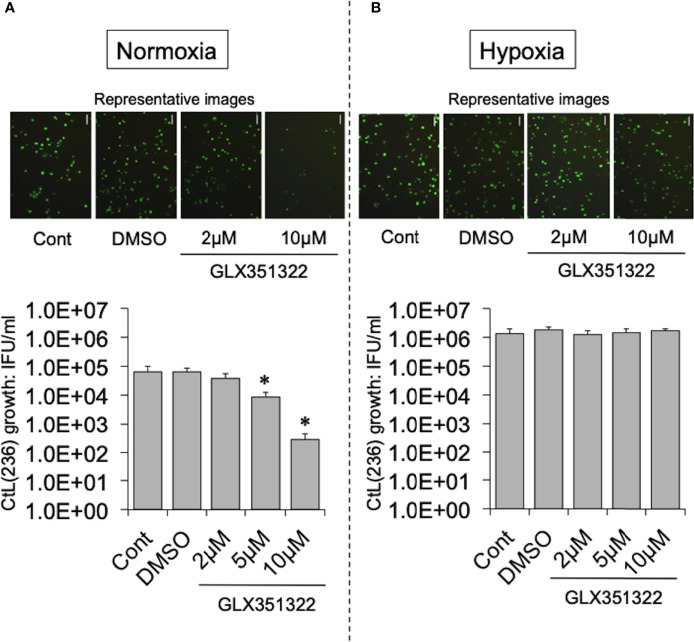
Effect of NOX4-specific inhibitor GLX351322 on the intracellular growth of green fluorescent protein (GFP)-expressing CtL2 (236) under normoxia **(A)** and hypoxia **(B)**. HEp-2 cells were infected at a multiplicity of infection (MOI) = 5 with GFP-expressing CtL2 (236) in the presence or absence of the drug, and cultured for 48 h. Representative images show inclusion bodies (green) formed in infected HEp-2 cells. Bars = 100 μm. The number of bacteria was calculated by inclusion-forming unit (IFU) assay of infected cells cultured for 48 h. Data show means ± SD obtained from at least three experiments. *p < 0.05 vs. the value for each control (Cont).

**Figure 3:**
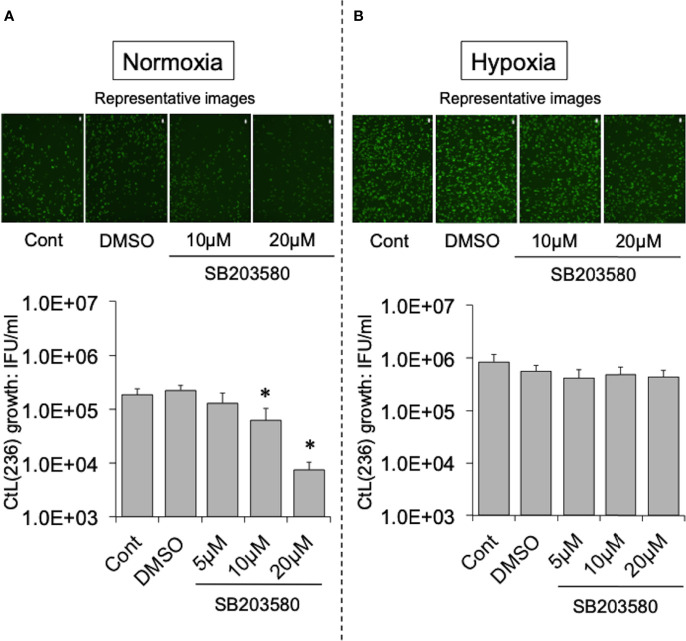
Effect of NOX4-downstream molecule p38MAPK-specific inhibitor SB203580 on the intracellular growth of GFP-expressing CtL2 (236) under normoxia **(A)** and hypoxia **(B)**. The HEp-2 cells were infected at MOI = 5 with GFP-expressing CtL2 (236) in the presence or absence of the drug, and cultured for 48 h. Representative images show inclusion bodies (green) formed in infected HEp-2 cells. Bars = 200 μm. The number of bacteria was calculated by IFU assay of infected cells cultured for 48 h. Data show means ± SD obtained from at least three experiments. *p < 0.05 vs. the value of each control (Cont).

To test this hypothesis, the effect of more specific NOX inhibitors (ML171 and GLX351322) on the growth of CtL2 under normoxia (21% O_2_) and hypoxia (2% O_2_) was compared. In contrast to hypoxia, both inhibitors significantly inhibited the growth of CtL2 under normoxia in a concentration-dependent manner (**Figure S2** and **Figure 2**). The growth of CtL2 was also significantly suppressed in NOX4-knockdown cells treated with small interfering RNA (siRNA) against NOX4 under normoxia (**Figure S3**). Thus, NOX4 clearly plays a crucial role in the growth of Ct under normoxia, but not under hypoxia. Next, the role of p38MAPK, which is a NOX4-related molecule (Corbi et al., 2013), on the growth of CtL2 was assessed using a p38MAPK-specific inhibitor, SB203580. Similar to the effects of ML171 and GLX351322, the presence of SB203580 significantly diminished the intracellular growth of CtL2 under normoxia, but not under hypoxia (**Figure 3**). Thus, as expected, our findings indicated that CtL2 relies on NOX4/p38MAPK signaling for its growth under normoxia, but not under hypoxia.

As supported by several studies (Irani, 2000; Basuroy et al., 2011; Lee et al., 2014; Ribeiro-Pereira et al., 2014; Beretta et al., 2020), NOX4-dependent ROS has an important role as second messengers associated with cellular survival under normoxia. The mechanism involves the stabilization of mitochondrial function, which is responsible for the stable supply of ATP, *via* cross-talk between NOX4 and Mt (Dan Dunn et al., 2015). The exposure of cells to ROS can activate p38MAPK, which is a NOX4-related molecule (Bedard and Krause, 2007). Thus, ROS may play an important role in the requirement of NOX/p38MAPK for the intracellular growth of CtL2 under normoxia.

The inhibitors ML171, GLX351322, and SB203580, however, had no effect on the growth of Ct under hypoxia. Unlike under normoxia, the energy source of Ct in low O_2_ conditions is ATP produced by glycolysis in the infected host cells following the activation of PI3K/AKT (Jerchel et al., 2014; Lavu et al., 2020; Thapa et al., 2020) In fact, several studies have reported that Ct activates PI3K, which regulates glycolysis, by phosphorylating AKT (Zou et al., 2019; Thapa et al., 2020; Huang et al., 2021).

### CtL2 Relies on Host-Cell Mt for its Growth Under Normoxia, but Not Under Hypoxia

**Figure 4:**
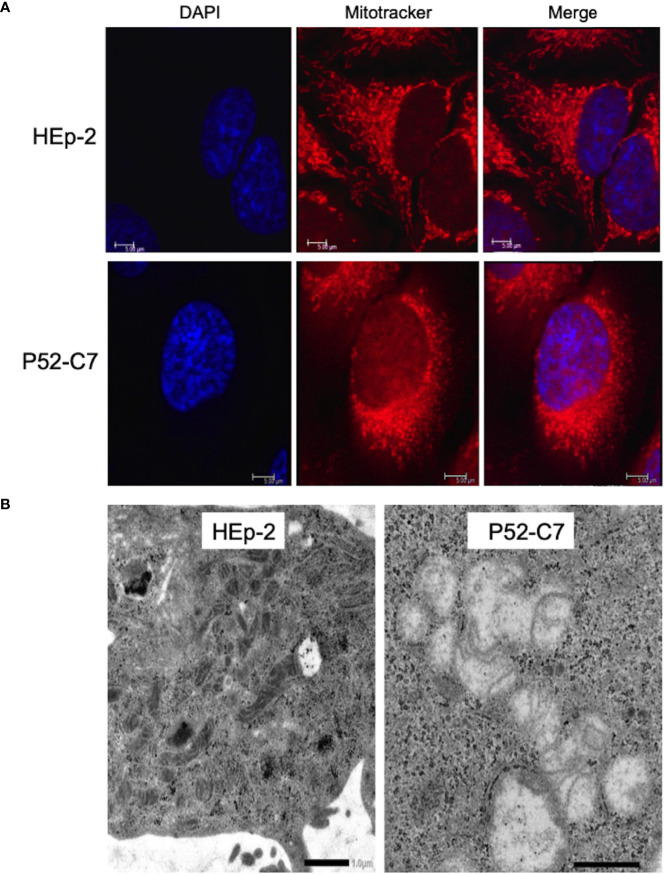
Morphological features of mitochondria (Mt) in HEp-2 cells with or without ethidium bromide exposure. **(A)** Representative confocal laser microscopy images showing the localization and morphology of Mt in Mt^d^-HEp-2 (P52-C7) cells and parental HEp-2 cells stained using MitoTracker (see *Methods*). Bars = 5 μm. **(B)** Representative transmission electron microscopy images showing the detailed morphology of Mt in Mt^d^-HEp-2 (P52-C7) cells and parental HEp-2 cells. Scale bars = 1 μm.

**Figure 5:**
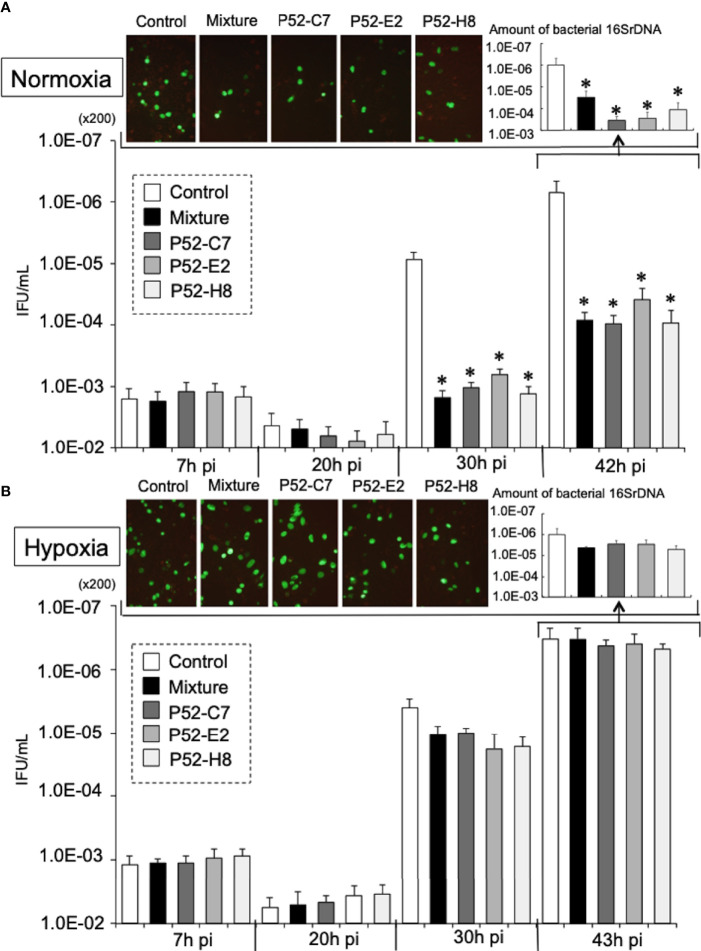
Growth kinetics of GFP-expressing CtL2 (236) in Mt^d^-HEp-2 (P52-C7, P52-E2, P52-H8) cells and parental HEp-2 cells under normoxia **(A)** and hypoxia **(B)**. The cells were infected with GFP-expressing CtL2 (236) at MOI = 5 and then cultured for 48 h under normoxia or hypoxia. Bacterial numbers were determined at 7, 20, 30, and 43 h by IFU assay and qPCR. Magnification for images, ×200. The quantities of chlamydial *16S rDNA* were normalized to that of *β-actin*. Data show the means ± SD obtained from at least three experiments. *p < 0.05 vs. the value of each control (white bar).

According to a previous report (Yu et al., 2007), Mt^d^-HEp-2 cells were successfully established by the passage of HEp-2 cells for 6 months in the presence of ethidium bromide (EtBr) at low concentration (50 ng/ml) with subsequent cloning (**Figure S4A**); five strains (P52-B3, P52-B10, P52-C7, P52-E2, P52-H8) were established. The mitochondrial genome consists of 37 genes, including tRNAs (Nicholls and Minczuk, 2014). The Mt^d^-HEp-2 cells that we generated had lost the *D-loop*, which is associated with replication of the mitochondrial genome, and *COXII*, which encodes a component of respiratory chain Complex IV (**Figure S4B**). Compared with HEp-2 cells without EtBr exposure, the amounts of NADH and NADPH significantly increased in the Mt^d^-HEp-2 cells under normoxia, indicating an activation of aerobic glycolysis, referred to as the Warburg effect (**Figure S4C**) (Vaupel and Multhoff, 2021). Confocal laser fluorescence imaging and transmission electron microscopy (TEM) observations revealed that in Mt^d^-HEp-2 cells, the Mt swelled (**Figure 4A**), corresponding to the observations in the previous report (Yu et al., 2007) (**Figure 4B**). Together, these findings showed that in Mt^d^-HEp-2 cells, the Mt became dysfunctional, indicating that this cell line was a useful tool for verifying the effects of Mt on the intracellular growth of Ct. Under normoxia, Ct growth was significantly inhibited in Mt^d^-HEp-2 cells; under hypoxia, there was no growth inhibition (**Figure 5**). Thus, under normoxia, Ct relies on functional Mt as a source of ATP, consisting with the previous studies showing the presence of crosstalk between Ct metabolism and mitochondria (Szaszák et al., 2011; Chowdhury and Rudel, 2017).

EtBr selectively binds to DNA and its accumulation causes mutations and deletions in DNA (Hayashi et al., 1994). The damage to DNA is more pronounced in mitochondrial DNA, whose repair mechanism is fragile, compared with genomic (nuclear) DNA. Thus, the application of EtBr can selectively induce cells with dysfunctional Mt (Yu et al., 2007; Luo et al., 2013). As expected, the Hep-2 cells serially passaged with EtBr exposure here showed morphological changes of the Mt and the Warburg effect. We therefore concluded that Mt^d^-HEp-2 cells were successfully established (Yu et al., 2007). Meanwhile, subculture of the cells in the absence of EtBr for >1 week decreased the amount of NADH and NADPH (data not shown), which showed restoration of normal mitochondrial function, indicating that not all of the Mt in the cells were completely dysfunctional. However, because the culture period of the infected cells without EtBr in our experiments was only 2 days, the effect of mitochondrial restoration was minimal.

Although no difference was observed under hypoxia, under normoxia, the growth of Ct in Mt^d^-HEp-2 cells was significantly decreased compared with that in the parental HEp-2 cells. This finding indicates that under normoxia, Ct relies on Mt as its source of ATP. We also found that NOX4/p38MAPK signaling, which is involved in the control of mitochondrial function (Bedard and Krause, 2007; Dan Dunn et al., 2015), plays a critical role in the intracellular growth of Ct under normoxia. Furthermore, it is evident that the amounts of ROS generated from NOXs is increased under normoxia compared with hypoxia (Basuroy et al., 2011; Lee et al., 2014; Ribeiro-Pereira et al., 2014; Beretta et al., 2020), and crosstalk between NOXs and Mt has been proposed to be a crucial mechanism for cellular survival (Fukai and Ushio-Fukai, 2020), as well as being responsible for maintaining a stable supply of ATP to Ct. Thus, under normoxia, Ct require functional Mt with the activation of NOX4/p38MAPK signaling, presumably *via* ROS as a second messenger. Crosstalk between NOX4 and Mt *via* p38MPAK may be crucial for supporting the intracellular growth of Ct in the presence of O_2_.

Under hypoxia, Ct obtains ATP from glycolysis by the activation of PI3K/AKT signaling (Zou et al., 2019; Thapa et al., 2020; Huang et al., 2021). We therefore speculate that Ct switches its energy source between Mt and glycolysis in response to change in the ATP-production site in the infected cells, which in turn depends on the cellular O_2_ concentration. We also expect that Ct modifies host cell signaling using effector molecules that are injected into the cytoplasm *via* the type III secretion system (Dai and Li, 2014). Meanwhile, further study is needed to verify this in detail and to establish the switching mechanism and effectors. Also, there was a limitation of the used cell line producing ATP mainly *via* glycolysis that should be addressed by additional experiments.

## Conclusions

In contrast to hypoxia, CtL2 requires functional Mt with NOX4/p38MAPK-mediated signaling for its growth under normoxia. Crosstalk between NOX4 and Mt *via* p38MAPK may be crucial for the growth of Ct under normoxia. These findings provide novel insight into the complicated biology and pathogenesis of *Chlamydia*.

## Methods

### Bacteria and Human Cells

Chlamydiae [CtL2 (L2 434/Bu) and CtD (D/UW3/CX)] and immortal human epithelial HEp-2 cells were used for this study. The bacteria were propagated into HEp-2 cells in Dulbecco’s Modified Eagle’s Medium (Sigma, Burlington, MA) containing 10% fetal calf serum and antibiotics (10 µg/ml gentamicin, 10 µg/ml vancomycin, and 1 µg/ml amphotericin B) at 37°C under normoxia, and then stored at −80°C, according to a previously described protocol (Thapa et al., 2020). CtL2 were transformed using green fluorescent protein (pGFP)::SW2 with a promoter (p) derived from *Neisseria meningitidis* replaced with p-CT236 (hypothetical gene) or p-CT267 (hypothetical gene), both of which can bind more efficiently to the ribosome (as determined using a ribosome binding site calculator) than the original promoter (Howard, 2011), according to the protocol for chlamydial transformation (Bauler and Hackstadt, 2014).

### Establishment of Mt^d^-HEp-2 Cells

As shown in **Figure S4A**, Mt^d^-HEp-2 cells (P52-B3, P52-B10, P52-C7, P52-E2, and P52-H8) were established by the passage of HEp-2 cells for 6 months in the presence of EtBr (50 ng/ml) with supplements [sodium pyruvate (100 μg/ml) and uridin (50 ng/ml)] and subsequent cloning with a limited dilution method, according to previous report (Yu et al., 2007). The dysfunctional state of Mt was confirmed by the loss of two genes (*D-loop* and *COXII*) from the mitochondrial genome by qPCR, increased amounts of NADH/NADPH (aerobic glycolysis, Warburg effect), and changed mitochondrial morphology (see below).

### Establishment of NOX4-Knockdown Cells

Transient NOX4-knockdown HEp-2 cells were established by 24-h transfection of cells with NOX4 siRNA (sc-41586; Santa Cruz Bio). Non-targeting scramble RNA (sc-37007; Santa Cruz Bio) was used as a control. Transfection of siRNA (or scramble RNA) into cells was performed with Lipofectamine™ RNAiMAX Transfection Reagent (Thermo Fisher), according to the manufacturer’s protocol. The amount of the complex brought into the culture system was 250 μl [Cont-siRNA (250) and NOX4-siRNA (250)] or 50 μl [Cont-siRNA (50) and NOX4-siRNA (50)] (see **Figure S3**).

### Assessing Inclusion-Forming Units

The number of infectious progeny (EB) was determined as IFU by counting chlamydial inclusion bodies formed in epithelial cells using a fluorescein isothiocyanate-conjugated anti-chlamydial monoclonal antibody specific to *Chlamydia* lipopolysaccharide (Denka Seiken, Tokyo, Japan), as described previously (Thapa et al., 2020). Cells were observed using an Olympus culture microscope, model CKX41.

### Infection and Culture

Cells were infected at an appropriate multiplicity of infection (MOI) with Ct (some expressing GFP as described above) or Cp, and then cultured in 10% FCS-RPMI medium with or without inhibitors of NOXs [DPI (anti-ROS: 0.4-10 nM), ML171 (anti-NOXs: 2-10 μM), GLX351322 (anti-NOX4: 2-10 μM), or SB203580 (anti-p38MAPK: 2.5-10 μM)] for 48 h under normoxia (21% O_2_) or hypoxia (2% O_2_). Hypoxia was established using a dedicated MIC-101 chamber (Billups-Rothenberg) to which mixed gas containing 2% O_2_, 5% CO_2_, and 93% N_2_ was supplied, as previously described (Thapa et al., 2020). O_2_ conditions were continuously monitored using an oxygen meter.

### Assessing Amounts of NADH and NADPH

The total amounts of NADH/NADPH in cultures were quantified by using the Cell Counting Kit-8 (Dojindo), according to the manufacturer’s protocol. The values were calculated from measurements of OD_450 nm_.

### Imaging of Mt

Cells were stained with MitoTracker^®^ Red CMXRos, and then fixed with 4% paraformaldehyde in phosphate-buffered saline (PBS), following the manufacturer’s protocol (Cell Signaling). The stained cells were observed using a confocal laser fluorescence microscope (TCSSP5, Leica) or a conventional fluorescence microscope (BZX800, Keyence). Furthermore, infected cells were fixed using 3% glutaraldehyde in PBS. The cells were immersed in alcohol for dehydration after washing with PBS and then embedded in Epon 813. Ultrathin sections were obtained and stained with lead citrate and uranium acetate, followed by TEM observation (JEM-1400Flash, JEOL Ltd.), as described previously (Matsuo et al., 2008).

### DNA and RNA Extraction

Total DNAs and RNAs were extracted from cells using an Instagene kit (Bio-Rad, Hercules) and High Pure RNA Isolation Kit (Roche), respectively, following the manufacturers’ protocols. The reverse transcription of total RNA to cDNA was performed with ReverTraAce qPCR RT Master Mix (Toyobo).

### Quantitative PCR

Amplifications of DNA or cDNA were quantified by CFX Connect (Bio-Rad) with SYBR Green (KOD SYBR qPCR Mix, TOYOBO) targeting *D-loop* (forward: 5′- CCT GTC CTT GTA GTA TAA AC -3′; reverse: 5′- TTG AGG AGG TAA GCT ACA T -3′) (Yu et al., 2007), *COXII* (forward: 5′- TTC ATG ATC ACG CCC TCA TA -3′; reverse: 5’- CGG GAA TTG CAT CTG TTT TTA -3’) (Yu et al., 2007), chlamydial *16S rDNA* (forward: 5′-CGG CGT GGA TGA GGC AT-3′; reverse: 5′-TCA GTC CCA GTG TTG GC-3′), or *β-actin* (forward: 5′- GAC CAC ACC TAC AAT GAG -3′; reverse: 5′- GCA TAC CCC TCG TAG GG -3′) (Ishida et al., 2014). The quantities of *D-loop*, *COXII*, and chlamydial *16SrDNA* were normalized to that of *β-actin*.

### Western Blotting

Cells were lysed in RIPA buffer containing 0.1% sodium dodecyl sulfate (SDS; Nacalai Tesque). The proteins in each sample were separated by 8% SDS-polyacrylamide gel electrophoresis. The separated proteins were transferred to a polyvinylidene difluoride membrane by semi-dry electroblotting using the Trans-Blot^®^ Turbo™ blotting system (Bio-Rad). After blocking with 3% skim milk in Tris-buffered saline and 0.1% Tween 20 (TBS-T), membranes were incubated with primary antibody [anti-NOX4 (Abcam): ×2000; anti-tubulin (Santa Cruz Bio): ×2000] overnight at 4°C. After washing with TBS-T, membranes were incubated with secondary antibody for 4–6 h at 4°C. After washing, membranes were developed with Clarity™ Western ECL substrate (Bio-Rad) and visualized using a Chemi Doc™ XRS (Bio-Rad).

### Statistical Analyses

Comparisons among group values were performed by using the Bonferroni/Dunn test. A *p*-value of < 0.05 was considered statistically significant.

## Data Availability Statement

The original contributions presented in the study are included in the article/**Supplementary Material**. Further inquiries can be directed to the corresponding author.

## Author Contributions

HY conceived and designed the study. JT, GY, KI, and TO performed the laboratory work. JT and HY analyzed the data. SN performed imaging. YF, HH, and HY established the GFP-expressing Ct. HY wrote the manuscript with revision by JT. All authors contributed to the article and approved the submitted version.

## Funding

This study was supported by grants-in-aid from the Japan Society for Scientific Research KAKENHI (Grant numbers: 16H05225 and 21H02726). The funder had no role in the study design, data collection and analysis, decision to publish, or preparation of the manuscript.

## Conflict of Interest

The authors declare that the research was conducted in the absence of any commercial or financial relationships that could be construed as a potential conflict of interest.

## Publisher’s Note

All claims expressed in this article are solely those of the authors and do not necessarily represent those of their affiliated organizations, or those of the publisher, the editors and the reviewers. Any product that may be evaluated in this article, or claim that may be made by its manufacturer, is not guaranteed or endorsed by the publisher.

## Supplementary Material

Supplementary Figure 1 Gene expression levels of NADPH oxidases (NOXs 1–4) **(A)** and effect of diphenyleneiodonium (DPI; 0.08–5 nM) on the growth of Chlamydiae **(B)**. Gene expression was assessed by quantitative (q) reverse transcription (RT) polymerase chain reaction (PCR), and expressed as a value of 2^Δ;Δ;−Ct^. The quantities of NOXs were normalized to that of *β-actin*. Data show averages from duplicate experiments. HEp-2 cells were infected at an appropriate multiplicity of infection [MOI; GFP-expressing CtL2 (267) MOI 5; CtD: MOI 5] and cultured for 72 h. The number of bacteria in cultures was then determined by qPCR assay. The quantities of chlamydial *16S rDNA* were normalized to that of *β-actin.* Data show means ± SD from at least three experiments. **p* < 0.05 *vs*. the value of each positive control (PC).

Supplementary Figure 2 Effect of ML171 (2 and 10 μM) on the intracellular growth of green fluorescent protein (GFP)-expressing CtL2 (236) in HEp-2 cells under normoxia **(A)** and hypoxia **(B)**. The HEp-2 cells were infected at MOI 5 with CtL2 (236), and then cultured for 48 h. Representative images show inclusion bodies (green) formed in infected HEp-2 cells. Bars = 100 μm. The number of bacteria was calculated by inclusion-forming unit (IFU) assay of infected cells cultured for 48 h. Data show means ± SD from at least three experiments. **p* < 0.05 *vs*. the value of each PC.

Supplementary Figure 3 The growth of GFP-expressing CtL2 (236) in NOX4-knockdown HEp-2 cells and confirmation of the NOX4 knockdown by western blotting. Transfected cells were infected with CtL2 (236) for 48 h, and the numbers of IFU were verified. The upper images show inclusion bodies in the knockdown cells [NOX4-siRNA (250)] with Cont-siRNA (250) used as the control (see *Methods*). The graph shows the number of IFU 48 h after infection. Data show means ± SD from five fields per well from a single experiment. **p* < 0.05 *vs*. each control [Cont-siRNA (250) or Cont-siRNA (50)]. The lower images show the amount of NOX4 protein in the knockdown cells (with α-tubulin used as a loading control).

Supplementary Figure 4 The establishment of Mt^d^-HEp-2 cells using ethidium bromide (EtBr). **(A)** Culture schedule of HREp-2 cells in the presence of EtBr. **(B)** The image (left) shows expression levels of the *D-loop* and *COXII* genes in the cells and the graph (right) shows the quantified amount of *COXII* gene expression. **(C)** The image (left) shows color changes indicating the amount of NADH and NADPH in the cells, and the graph (right) shows the amounts of NADH/NADPH in the culture of Mt^d^-HEp-2 cells. The controls were medium and parental (non-EtBr-treated) HEp-2 cells. **p* < 0.05 *vs*. parental HEp-2 cells.

## Abbreviations

Ct, *Chlamydia trachomatis*; Cp, *Chlamydia pneumoniae*; Mt, mitochondria; NOX, NADPH oxidase; DPI, diphenyleneiodonium; AKT, protein kinase B; EB, elementary body; ROS, reactive oxygen species; q, quantitative; RT, reverse transcription; PCR, polymerase chain reaction; EtBr, ethidium bromide; TEM, transmission electron microscopy; GFP, green fluorescent protein; TBS-T, Tris-buffered saline and 0.1% Tween 20.
