# Supplementary figures and images for: Chlamydia trachomatis Requires Functional Host-Cell Mitochondria and NADPH Oxidase 4/p38MAPK Signaling for Growth in Normoxia
Source: Front Cell Infect Microbiol. 2022 May 26;12:902492. doi: 10.3389/fcimb.2022.902492 (PMC9199516; doi:10.3389/fcimb.2022.902492)

A

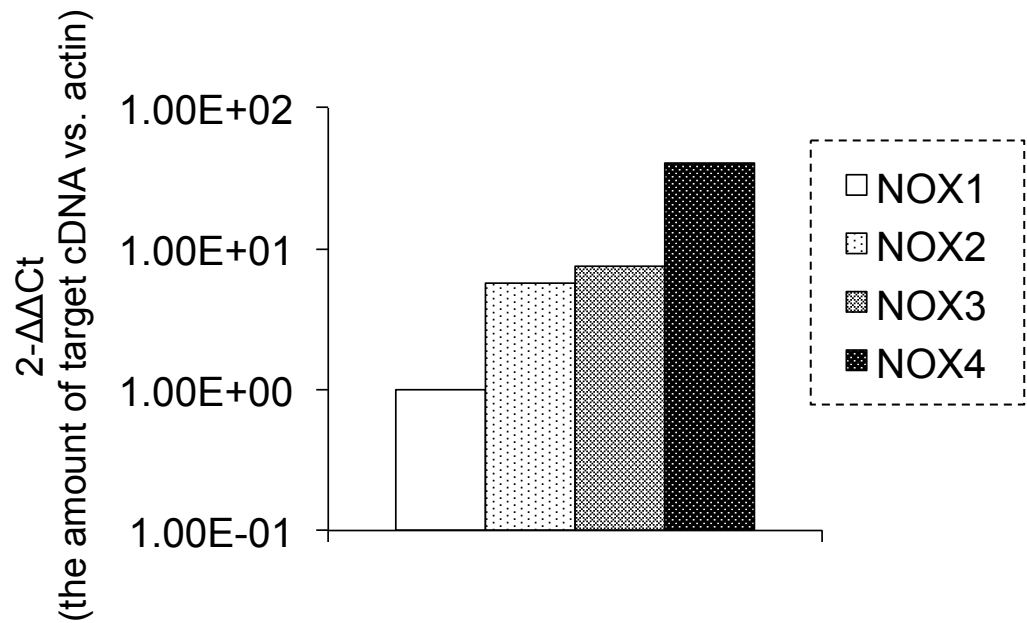

B

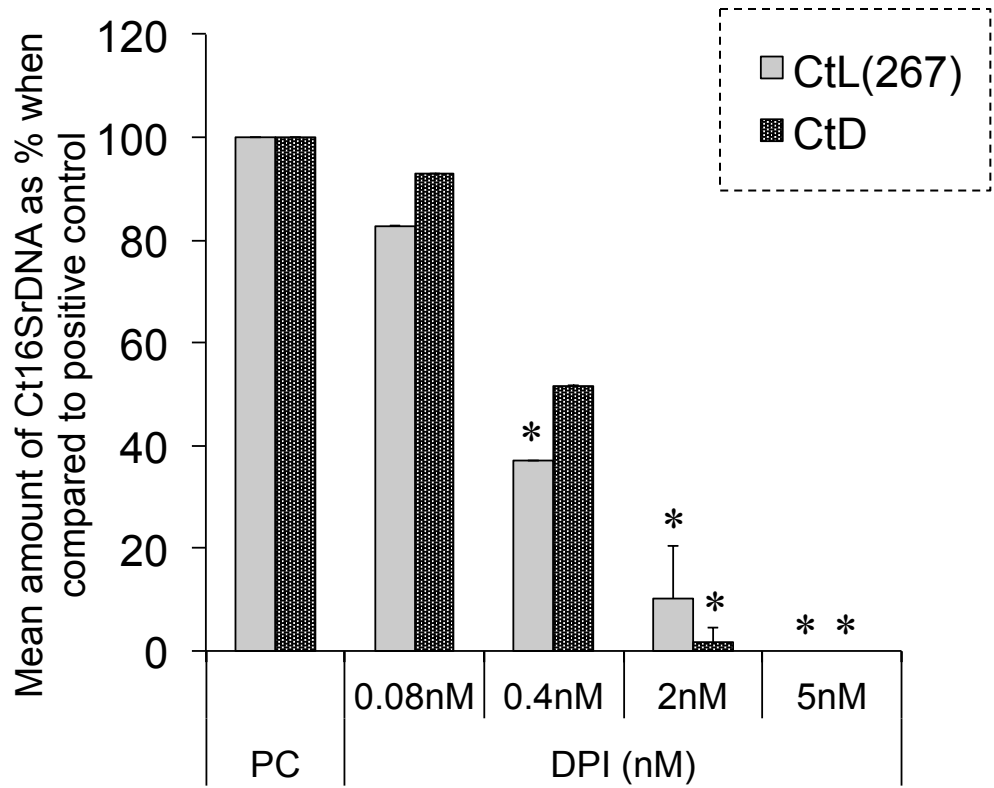

Supplement: Supplementary Figure 1 — Gene expression levels of NADPH oxidases (NOXs 1–4) (A) and effect of diphenyleneiodonium (DPI; 0.08–5 nM) on the growth of Chlamydiae (B). Gene expression was assessed by quantitative (q) reverse transcription (RT) polymerase chain reaction (PCR), and expressed as a value of 2Δ;Δ;−Ct. The quantities of NOXs were normalized to that of β-actin. Data show averages from duplicate experiments. HEp-2 cells were infected at an appropriate multiplicity of infection [MOI; GFP-expressing CtL2 (267) MOI 5; CtD: MOI 5] and cultured for 72 h. The number of bacteria in cultures was then determined by qPCR assay. The quantities of chlamydial 16S rDNA were normalized to that of β-actin. Data show means ± SD from at least three experiments. *p < 0.05 vs. the value of each positive control (PC). [file DataSheet_1.pdf]

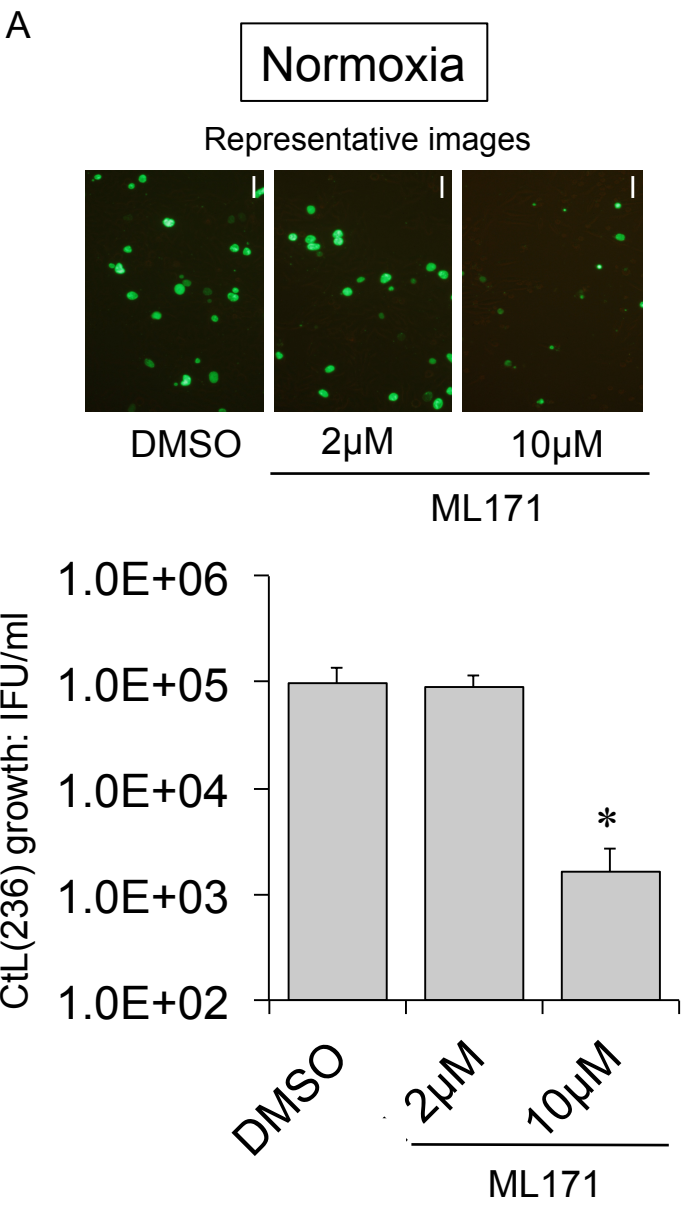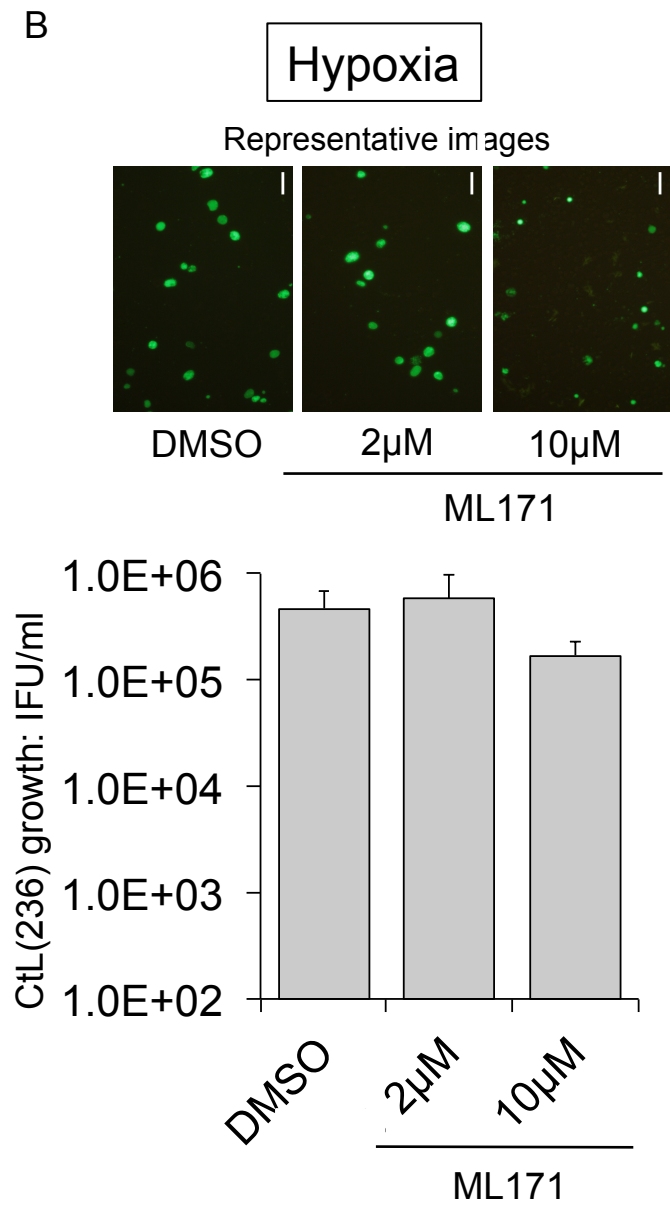

Supplement: Supplementary Figure 2 — Effect of ML171 (2 and 10 μM) on the intracellular growth of green fluorescent protein (GFP)-expressing CtL2 (236) in HEp-2 cells under normoxia (A) and hypoxia (B). The HEp-2 cells were infected at MOI 5 with CtL2 (236), and then cultured for 48 h. Representative images show inclusion bodies (green) formed in infected HEp-2 cells. Bars = 100 μm. The number of bacteria was calculated by inclusion-forming unit (IFU) assay of infected cells cultured for 48 h. Data show means ± SD from at least three experiments. *p < 0.05 vs. the value of each PC. [file DataSheet_2.pdf]

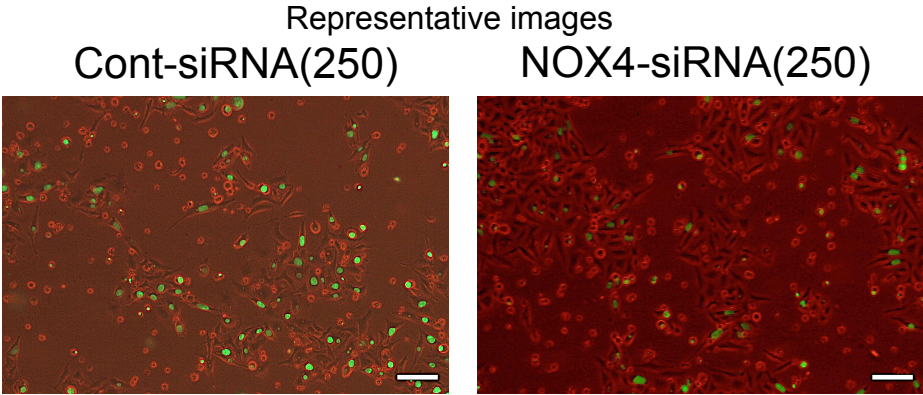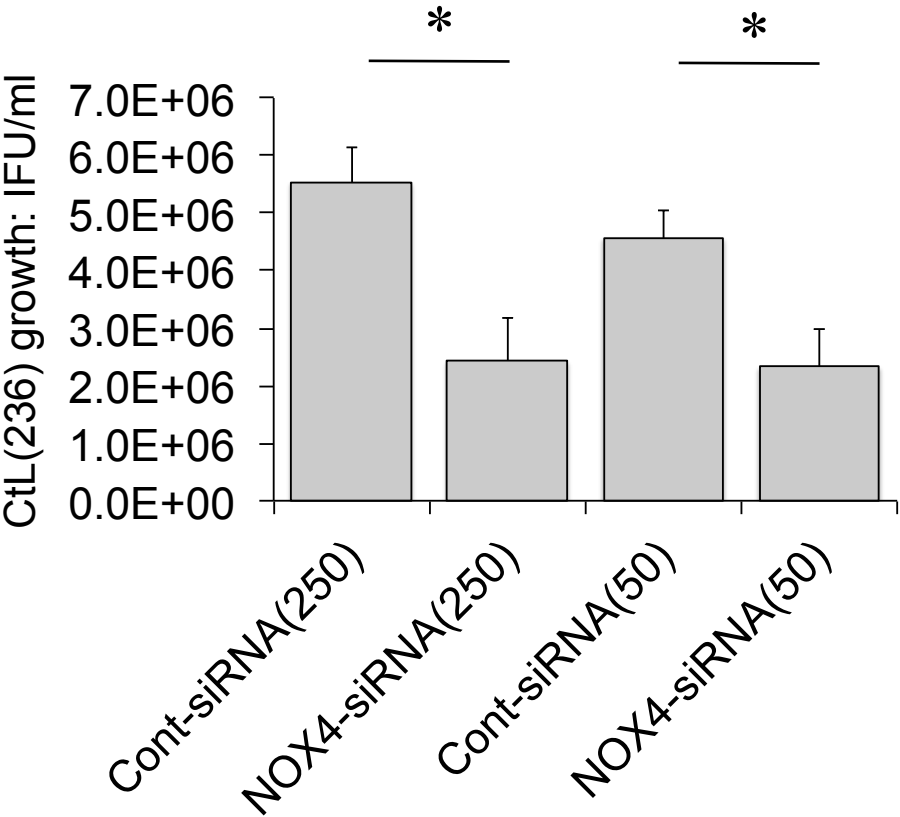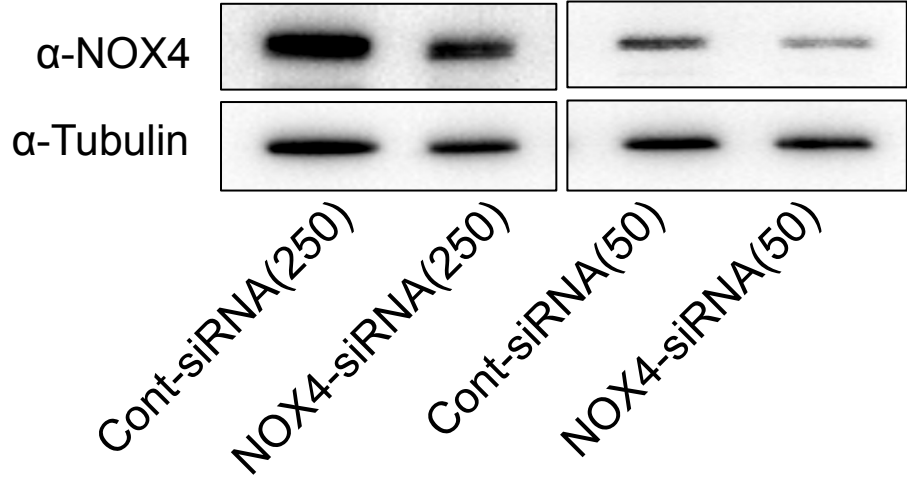

Supplement: Supplementary Figure 3 — The growth of GFP-expressing CtL2 (236) in NOX4-knockdown HEp-2 cells and confirmation of the NOX4 knockdown by western blotting. Transfected cells were infected with CtL2 (236) for 48 h, and the numbers of IFU were verified. The upper images show inclusion bodies in the knockdown cells [NOX4-siRNA (250)] with Cont-siRNA (250) used as the control (see Methods). The graph shows the number of IFU 48 h after infection. Data show means ± SD from five fields per well from a single experiment. *p < 0.05 vs. each control [Cont-siRNA (250) or Cont-siRNA (50)]. The lower images show the amount of NOX4 protein in the knockdown cells (with α-tubulin used as a loading control). [file DataSheet_3.pdf]

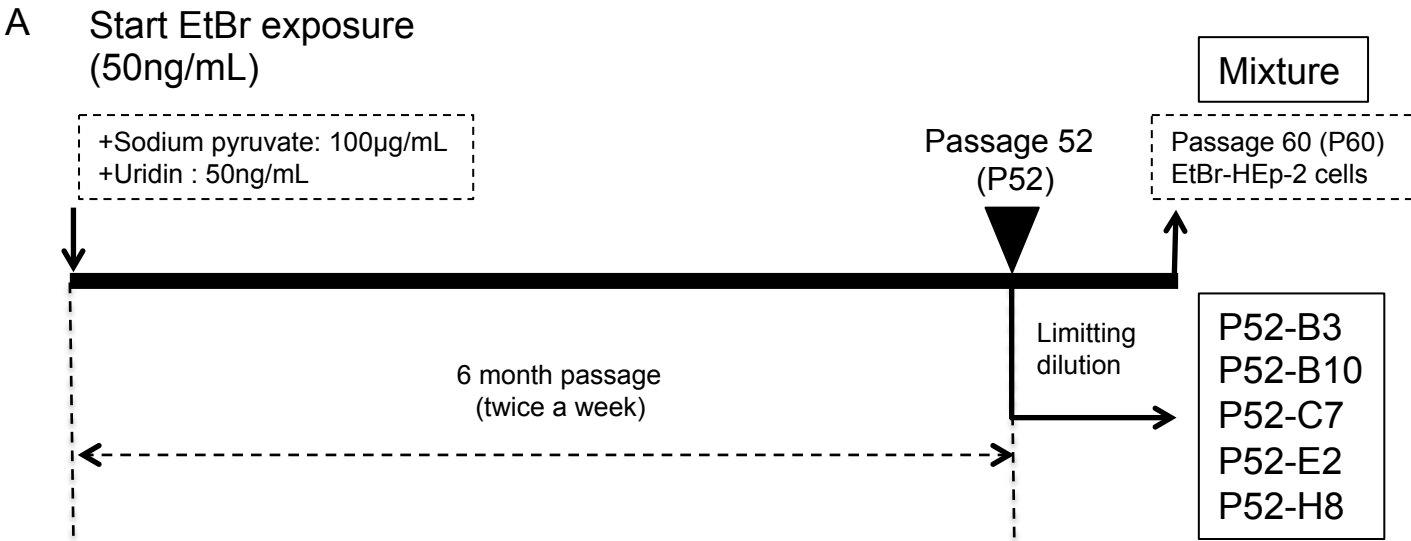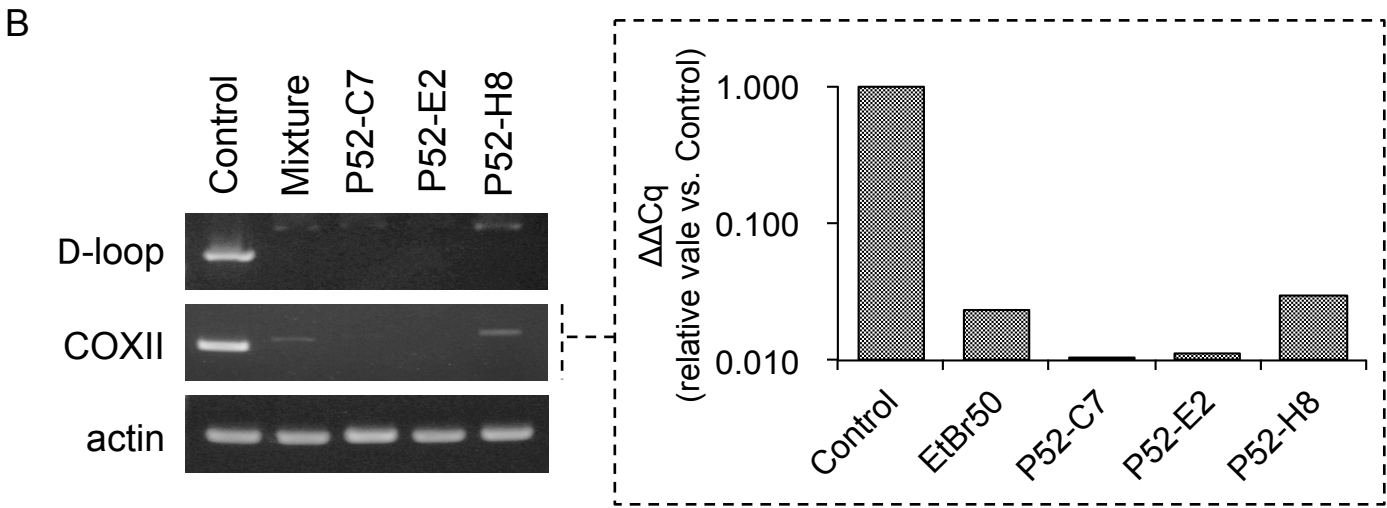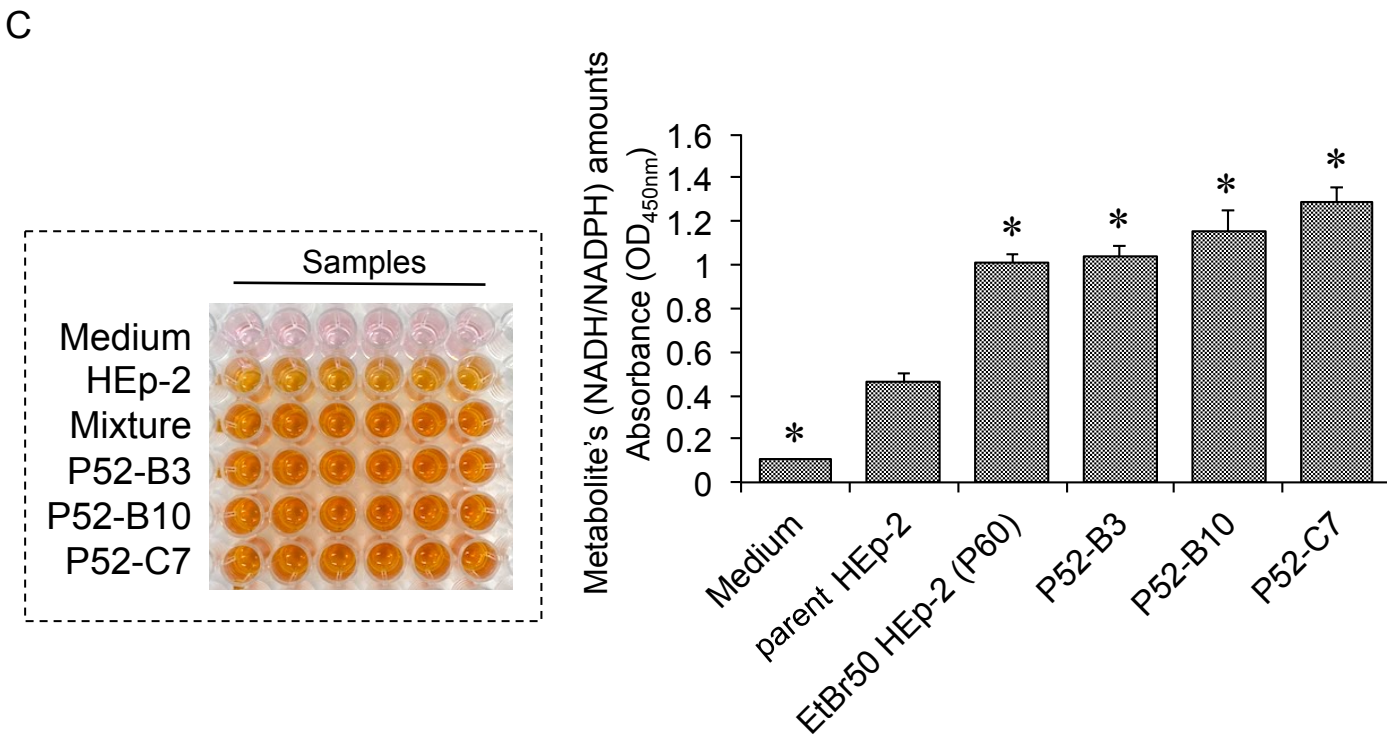

Supplement: Supplementary Figure 4 — The establishment of Mtd-HEp-2 cells using ethidium bromide (EtBr). (A) Culture schedule of HREp-2 cells in the presence of EtBr. (B) The image (left) shows expression levels of the D-loop and COXII genes in the cells and the graph (right) shows the quantified amount of COXII gene expression. (C) The image (left) shows color changes indicating the amount of NADH and NADPH in the cells, and the graph (right) shows the amounts of NADH/NADPH in the culture of Mtd-HEp-2 cells. The controls were medium and parental (non-EtBr-treated) HEp-2 cells. *p < 0.05 vs. parental HEp-2 cells. [file DataSheet_4.pdf]
